# Acceptability of Vegetable Fortified *Ugali* in Sub-Saharan Africa

**DOI:** 10.3390/nu13103405

**Published:** 2021-09-27

**Authors:** Zixuan Cai, Xin Meng, Dennis Nyirenda, Wilson Mandala, Xiaoyun Li, Dong Yang

**Affiliations:** 1 Beijing Key Laboratory of Functional Food from Plant Resources, College of Food Science & Nutritional Engineering, China Agricultural University, 17 East Tsinghua Rd., Beijing 100083, China; SY20183060967@cau.edu.cn (Z.C.); s20203061007@cau.edu.cn (X.M.); 2 Xinghua Industrial Research Centre for Food Science and Human Health, China Agricultural University, Xinghua 225700, China; 3 College of International Development & Global Agriculture, China Agricultural University, 17 East Tsinghua Rd., Beijing 100083, China; dennyirenda@yahoo.co.uk (D.N.); xiaoyun@cau.edu.cn (X.L.); 4 Academy of Medical Sciences, Malawi University of Science and Technology, Thyolo BT3, Malawi; wmandala@must.ac.mw

**Keywords:** fortification, *ugali*, sensory evaluation, vegetable, sub-Saharan Africa

## Abstract

Corn flour-based porridge like dough, *ugali*, is the staple food of low-income population in sub-Saharan Africa. Lack of vitamin A, carotenoids, and dietary fibers brings about serious health issues to this population. In this study, vegetables including bok choy, broccoli, cabbage, carrot, Chinese onion stalk (C_onion), mushroom, are added during the cooking of *ugali*, as nutritional supplements. The freeze-dried powder of each vegetable was used for its long storage, stable nutrients, and similar particle size. Sub-Saharan African assessors were trained and sensory evaluated the six different vegetable fortified *ugali* with the plain, unfortified as the control on five attributes. The plain *ugali* was indistinguishable with the C_onion stalk fortified in color, with the carrot and C_onion stalk fortified in odor, with all vegetables (except broccoli and mushroom) fortified *ugali* in taste, with carrot and C_onion stalk fortified in granularity, and with cabbage, carrot, C_onion stalk fortified in viscosity. Preference ranking analysis showed that the C_onion stalk fortified *ugali* is even more favorably preferred than the plain, unfortified *ugali*, probably due to the umami components in C_onion that serve as the taste enhancer. This study indicates that Chinese onion stalk is a potential vegetable supplement to population in the sub-Saharan Africa.

## 1. Introduction

**Figure 1:**
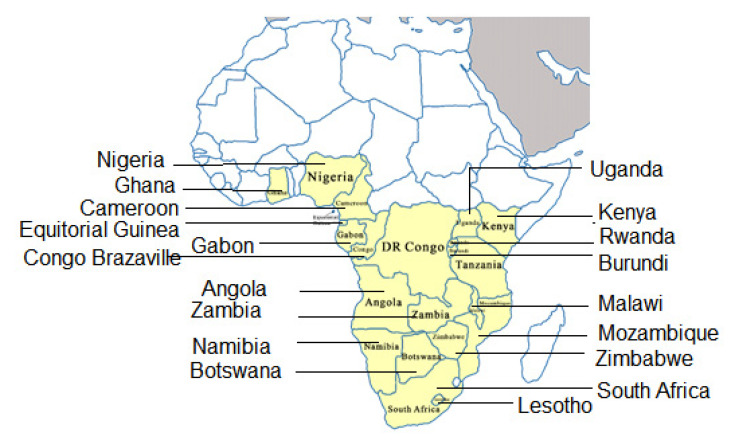
*Ugali* consumption in Africa. Countries in yellow are those that consume *ugali* as the main staple food; countries represented in white are those that consume *ugali* as one of several other staple foods.

In several sub-Saharan African countries, corn, cassava and sorghum are common crops whose yields merely satisfy local demands [1]. These crops are often ground into powder, mixed with water, boiled to cooked, and served as the staple food in a number of sub-Saharan African countries (Figure 1). *Ugali* is a stiff, starchy porridge made from flour of various crops which is prepared by gradual mixing of the flour with boiling water. When the flour-boiling water mixture is allowed to simmer further, the starch-rich slurry transforms into a viscous porridge as a result of gelatinization of the starch [2]. Depending on the country, *ugali* is usually served with side dishes of stews containing various relish like fish, legume, cooked green leafy vegetables, beef, sour milk or other sources of protein [3,4]. Although the *ugali* itself is known to be extremely low in terms of nutrition content, it is the quality and quantity of these side dishes that can potentially improve their nutrition value [4]. However when *ugali* is served with relish which is also low in nutrition value as is the case in most rural areas in sub-Saharan African countries, then the combined meal still remains of low caloric and nutritional value [4].

There are many names of this food according to the countries, regions, even tribes. It is most widely called *ugali*, which is also named as *bugali*, *gari*, *nshima*, *pap*, etc. in different countries (Table 1). Here we refer it to *ugali* as it is the name by which it first appeared in a nutritional study 33 years ago [5]. Normally, *ugali* is made with only white corn flour, but when the corn has a bad harvest, sorghum or cassava powder would be used instead to make *ugali*. Acting as the staple food for 250 million African people, if cassava is used in making *ugali* instead of corn, it seriously lacks zinc, iron, vitamin A and proteins [6,7], and contains cyanides that would cause nervous system damage to 5% African children after long term ingestion [8]. The low content of tryptophan and lysine in corn also renders insufficient protein in terms of nutrition need. Previous study revealed that after long-term ingestion of *ugali* as the only food, all the experimental rats displayed liver diseases to varying degrees [9]. These were mostly diffuse hepatic enlargement and extensive nodular cirrhosis caused by fat changes. In all, long term and sole intake of only *ugali* as the staple food introduces nutritional deficiency related health issues. One way to solve the undernutrition caused by intake of *ugali* as the sole staple food may commence with corn cultivation that trying to improve its nutrition content. Previous study shows that for the crops (mainly beans, rice, millet, cassava and corn) cultivated in South Asia, sub-Saharan Africa and Latin America, nutrition enhancement in minerals and carotene could apparently help suppress the symptom of iron and vitamin A deficiency for local people. Meanwhile, their physical, mental and cognitive abilities were significantly improved [10]. Promotion of vitamin A fortified sweet potato in Uganda and Mozambique showed that children and women in Uganda who consumed fortified sweet potato had a significant increase in vitamin A intake, while the Mozambican children also had a significant increase not only in vitamin A but also other vitamins [11,12]. However, it has proven to be a challenge to persuade the local people in some countries, especially the elderly to adopt these nutrition-enhanced crops when they are so used to either cultivating and/or consuming the old varieties that they are familiar with while they are unsure of the crop yield of the nutrition enhanced-varieties [13,14]. However, a recent study has reported much improved acceptability of these fortified varieties in a number of African countries [15].

**Figure 2:**
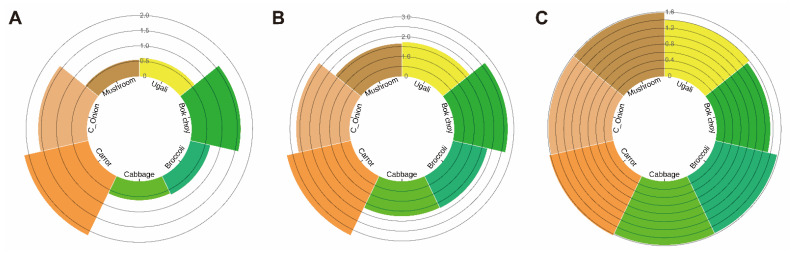
Nutrition value of 100 g different vegetable fortified *ugali*. (**A**) is the vitamin A content in 100 g vegetable fortified *ugali* (μg RAE/100 g) and (**B**) is the carotenoids content in 100 g vegetable fortified *ugali* (μg/100 g). The *y*-axis of (**A**,**B**) is in the log_10_ range. (**C**) is the dietary fiber content in 100 g vegetable fortified *ugali* (g/100 g). The nutrition value was calculated as 20 g of each vegetable fortified into 100 g corn flour *ugali*, and this figure shows the nutrition content of 100 g final fortified *ugali*. The original nutrition value of each vegetable was obtained from the U.S. Department of Agriculture, Food Data Central.

Another way to solve the nutrients deficiency in the staple food *ugali* is to add vegetables or fruits in *ugali*. Forsido et al. had tried adding Ethiopia banana to this Africa staple food, finding that the content of calcium and dietary fiber increased, so it was considered to have the potential to be applied in preparing this staple food [16]. When one considers adding fruits and vegetables to *ugali* for nutritional value increasing purposes, vitamin A or pro-vitamin A (β-carotene and carotenoids) should be considered with high priority as aforementioned. Bok choy, carrots, and cabbage have been supplied in meals to improve the total-body vitamin A pool size [17]. Broccoli, the Chinese onion (C_onion, *Allium schoenoprasum*), and mushroom (*Lentinus edodes*) are not only rich in vitamin A or carotenoids but also may add favorable flavors to *ugali* [18,19,20]. Dietary fiber has been proven beneficial through nourishing the gut microbiota [21,22]. Three types of flour paste from white corn (maize) can be used for making *ugali* depending on the how the white corn is processed [23]. Whole maize flour is produced from milling the dry maize without removing bran and is hence rich in fiber. Dehulled flour involved dehulling and winnowing to remove the bran and then milling the rest to produce the flour. Fermented flour, which is the most common, involved soaking (partially fermenting the maize grits for a day or two), washing and sun drying the maize grits before milling. Potentially, nutrients of dietary fiber are lost during the dehulling and winnowing stage when bran is removed [24] Fortification of watermelon pulp to another maze based food *Mageu* was tried, however, fruit fortification does not supply dietary fiber as vegetables do [25]. Thus, addition of dietary fiber through vegetable fortification, such as the Chinese onion stalk, is beneficial for the sub-Saharan population [26]. In this study, we chose bok choy, broccoli, cabbage carrot, Chinse onion (the stalk, C_onion), mushroom as the supplementary vegetables, for their rich content in either vitamin A, carotenoids, dietary fiber or both (Figure 2).

Freeze drying of vegetables not only enhances storage stability, reduces transportation weight, maximally maintains micro-nutrients, but also enables grinding of vegetables to powders that allows fortification into *ugali* [27,28]. The present study mainly focused on the sensory acceptability of freeze-dried vegetable powder fortified *ugali* to population with *ugali* as the staple food, as an evaluation of the feasibility of promoting vegetable fortified *ugali* in sub-Saharan Africa.

## 2. Materials and Methods

### 2.1. Materials

White corn flour was purchased from the supermarket for international students at the west campus of China Agricultural University. The freeze-dried vegetable of bok choy (*Brassica rapa* subsp. *chinensis*), broccoli, cabbage, carrot, C_Onion, mushroom (Xianggu, *Lentinus edodes*) were purchased from Jiangsu QingGu Foods Co., Ltd. (Xinghua, Jiangsu Province, China).

### 2.2. Preparation of Vegetables Foritfied Ugali

The freeze-dried vegetable was ground to the same particle (100 mesh) size before use. The white corn flour of 100 g was added gradually to 200 mL of boiling water and kept stirring all the time. Based on the nutritional value of each vegetable, 20 g could meet the daily requirement of nutritional intake of vitamin A, carotenoids, and dietary fiber. For vegetable fortified *ugali*, 20 g of each vegetable power was added during the stirring of *ugali* cooking, respectively. Heating and stirring was stopped until the corn flour was thoroughly cooked, and each cooked *ugali* was chilled before applied to sensory evaluation.

### 2.3. Sensory Evaluation

Sensory analysis of *ugali* was principally the same as previously performed on soymilk and chickpea [29,30,31]. The evaluation was conducted under white light and standardized conditions at the China Agricultural University, and each assessor was assigned a consent form prior to taking part in the study. After reading the consent form, assessors were asked if they understood the purpose and objectives of this study, procedures for assessing the cooked *ugali*, confidentiality of respondents and data, and the right to withdraw. Signed consent forms were collected from the assessors for record keeping purposes. After exclusion of assessors who were allergic to the abovementioned vegetables, a total of 24 assessors (international students in China Agricultural University from sub-Saharan African countries, 16 males and 8 females, aged 24–42 years old) were selected. The culinary traditions for *ugali* consumption in these countries are similar.

A “questionnaire” was provided to each assessor and they were requested to fill their gender, age and nationality (optional). Assessors were allowed to test 8 *ugali* samples in two sessions, 4 samples in each session and there was a 10 min break between sessions. In one session, assessors were required to clean their mouth with drinking water before and after assessing each sample, and take a break for 10 s between two samples. In each session, one plain *ugali* sample (with no vegetable addition) and three other vegetable fortified samples were offered with random sample codes (271, 472, 374, and 881 for the first session, 574, 331, 798, and 358 for the second session) and the content of the sample information was kept unknown to assessors. The samples were presented in small, transparent plastic containers, marked with the corresponding code, served with a spoon and a cup of drinking water. For one *ugali* sample, assessors evaluated five attributes including: color, odor, taste, granularity and viscosity. Assessors used a nine-point hedonic scale (from −4 = disgusting, 0 = neutral, to 4 = yummy). Finally, assessors were asked to rank the samples in order of preference (1 = least preferred, 8 = most preferred). The questionnaire was collected and data was processed.

### 2.4. Data Analysis

Sensory evaluation data of five attributes for two repeats of plain *ugali* were combined, and sample differences on each attribute were analyzed with least significant difference (LSD) test at a significance level of α = 0.05 with SPSS Statistic 26.0 (IBM Corp., Armonk, NY, USA). The violin plots were plotted with the GraphPad Prism (version 8.0, GraphPad Software Inc, La Jolla, CA, USA).

For median caregiver sensory rating, data of five attributes were compared by non-parametric Friedman’s two-way analysis of variance by ranks (*p* < 0.05) [29].

For preference ranking analysis, the sum of the rank for each sample was calculated, and that of the two blank groups was averaged. The ranking data was analyzed by Friedman test at a significance level of α = 0.01 to evaluate significant differences among all samples [32]. The test statistic *F* is calculated in Equation (1) where *R_i_* is the sum of ranks for sample *i*; *n* and *K* are the number of valid assessors and samples, respectively.

(1) $$F\;=\;\frac{12}{nK\left(K+1\right)}\cdot (R_{1}^{2}+R_{2}^{2}+\cdots +R_{K}^{2})-3n\left(K+1\right)$$

The test statistic *F* exhibits an asymptotic Chi-square distribution with *K*−1 degrees of freedom. At the α significance level, there are significant differences among all samples if *F* ≥ $\chi_{K-1;\alpha }^{2}$, where $\chi_{K-1;\alpha }^{2}$ is the α quantile of the Chi-square distribution with *K*−1 degrees of freedom. The significant differences between ranking data of two samples were analyzed with LSD test with SPSS Statistic 26.0 at a significance level of α = 0.05.

## 3. Results

Generally, the sensory evaluation process can be divided into two steps. The assessors were allowed to observe and smell the *ugali* samples before they try to taste them. And the initial observation and smelling of the sample profoundly impact personal willingness to taste. Thus, sensory evaluation data are divided into pre-tasting and tasting part for discussion.

### 3.1. Pre-Tasting Senseory Evaluaiton of Differently Fortified Ugali

**Figure 3:**
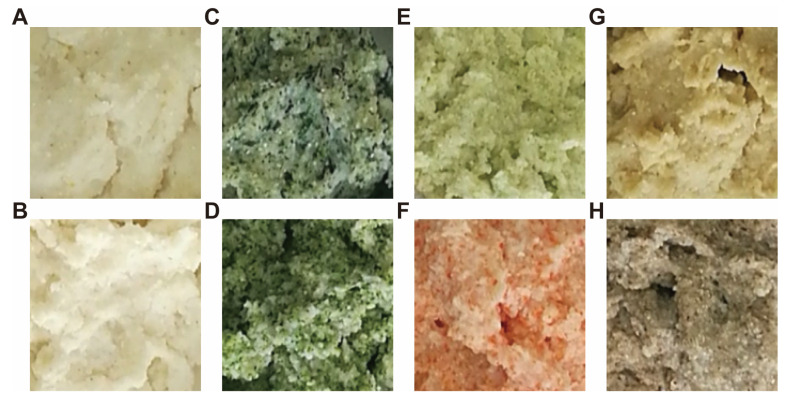
Images of differently fortified *ugali*. Images were taken right before different *ugali* presented to the accessors and in a close view that the color and granules could be clearly observed. (**A**,**B**) are the plain *ugali* with no vegetable addition. (**C**–**H**) are *ugali* fortified with bok choy, broccoli, cabbage, carrot, Chinese onion stalk, and mushroom, respectively.

The plain, corn flour-based *ugali* exhibited a white color with tint yellow (Figure 3A, B). Addition of different freeze-dried vegetable powder apparently altered the color of *ugali*. Specifically, addition of bok choy, broccoli, and cabbage endowed *ugali* a green color, with decreasing extent. That is, bok choy endowed *ugali* a dark green color (Figure 3C), broccoli endowed *ugali* a green color (Figure 3D), and cabbage endowed *ugali* a light green color (Figure 3E). On the other hand, addition of carrot endowed *ugali* a pink color (Figure 3F), addition of Chinese onion stalk endowed *ugali* a tint yellow color (Figure 3G), while addition of mushroom endowed *ugali* a brown color (Figure 3H).

**Figure 4:**
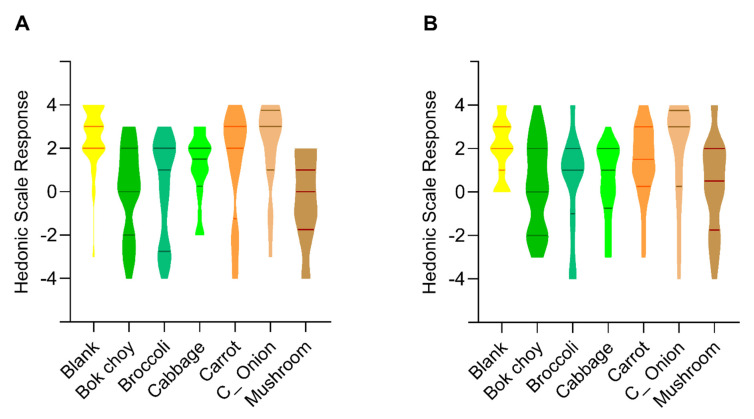
Pre-tasting sensory evaluation of differently fortified *ugali*. (**A**) violin plot of the sensory evaluation of color of differently fortified *ugali*; (**B**) violin plot of the sensory evaluation of odor of differently fortified *ugali*. The x-axis title indicates the vegetable powder added in corresponding *ugali*, and lines in each violin plot are the upper quartile, median, and lower quartile line, respectively.

Sensory evaluation of the color by assessors discriminated the color of plain *ugali* with these fortified with bok choy, broccoli, cabbage, carrot, and mushroom (Figure 4A, *p* < 0.05), but not with *ugali* fortified with Chinese onion stalk. For *ugali* added with bok choy, broccoli and cabbage, there is no significant difference among them in presenting the green color. Interestingly, there is also no significant difference between *ugali* fortified with cabbage, carrot, and Chinese onion stalk. What’s more, *ugali* fortified with mushroom looks apparently less preferable by color from these fortified with cabbage, carrot, Chinese onion stalk, and the plain *ugali* (*p* < 0.05), but not with *ugali* fortified with bok choy or broccoli.

Sensory evaluation of the odor by the assessors discriminated the odor of plain *ugali* with these fortified with bok choy, broccoli, cabbage, and mushroom that showing less preferable hedonic scale (Figure 4B, *p* < 0.05), but not these fortified with carrot and Chinese onion stalk. Meanwhile, there is no significant difference between carrot fortified *ugali* and these fortified with bok choy, broccoli, cabbage, Chinese onion stalk and mushroom. On the other hand, Chinese onion stalk fortified *ugali* distinguished itself from every other sample (*p* < 0.05) except carrot fortified- and the plain *ugali*.

### 3.2. Tasting Sensory Evaluation of Differently Fortified Ugali

**Figure 5:**
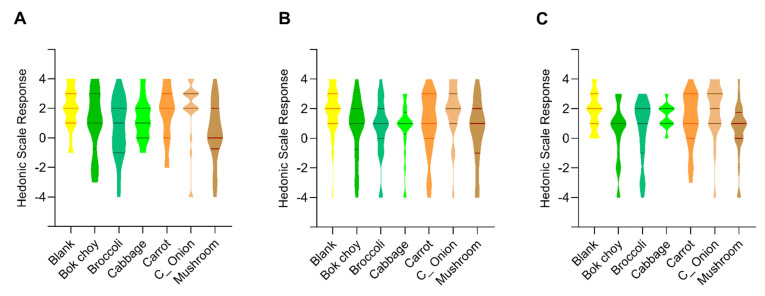
Tasting sensory evaluation of differently fortified *ugali*. (**A**) violin plot of the sensory evaluation of taste of differently fortified *ugali*; (**B**) violin plot of the sensory evaluation of granularity of differently fortified *ugali*. (**C**) violin plot of the sensory evaluation of viscosity of differently fortified *ugali*. The *x*-axis title indicates the vegetable powder added in corresponding *ugali*, and lines in each violin plot are the upper quartile, median, and lower quartile line, respectively.

While the plain, corn flour based *ugali* has no special flavor, different vegetables addition gives different flavor to *ugali* that may or may not satisfy its consumers. The taste was sensory evaluated to examine the catering to the potential consumers of each vegetable fortified *ugali*. Among all the vegetable added *ugali*, only the broccoli and mushroom added *ugali* exhibited significant less favorable responses in taste than the plain *ugali* (Figure 5A, *p* < 0.05). Meanwhile, the taste is indistinguishable from broccoli added *ugali* and bok choy, cabbage, carrot, mushroom fortified *ugali*. And the taste of mushroom added *ugali* is indistinguishable from bok choy, broccoli, and cabbage added *ugali*.

Although all vegetable powders were ground to the same size, they were all larger than the fine corn flour particles used in this study. The differences in the vegetable textures may introduce a variable in the mouth feeling that influences consumer experience. The granularity was sensory evaluated to exam the acceptance to the potential consumers of each vegetable fortified *ugali*. Among them, bok choy, broccoli, cabbage, and mushroom added *ugali* is significantly less preferred from the plain *ugali* as assessors evaluated (Figure 5B, *p* < 0.05). And there is no distinguishable granularity difference between the plain, carrot and Chinese onion stalk added *ugali*.

*Ugali* itself is a half-solid, half-liquid, viscous, porridge like food. Fortification with different vegetables may impact its viscosity that influences consumer mouth feeling. The viscosity was sensory evaluated to exam the acceptance to potential consumers of each vegetable added *ugali*. The cabbage, carrot, and Chinese onion stalk fortified *ugali* exhibited no differences in the mouth viscosity compared with the plain *ugali*. While there is a significant sensible viscosity difference between the plain *ugali* and the bok choy, broccoli, and mushroom added *ugali* (Figure 5C, *p* < 0.05).

### 3.3. Caregive Acceptability and Preference Ranking of Differently Fortified Ugali

**Figure 6:**
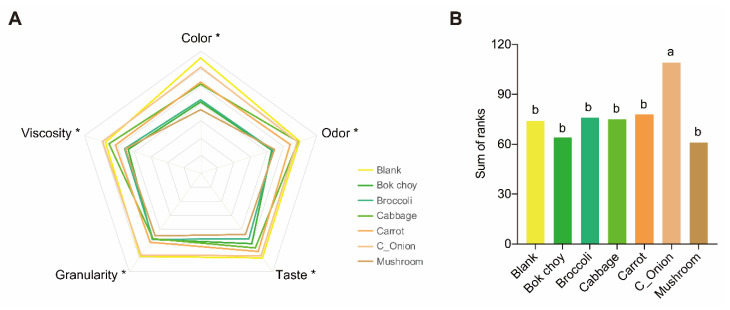
Caregiver evaluation and Preference ranking of differently fortified *ugali*. (**A**) caregiver evaluation of differently fortified *ugali*. * indicates significant difference between fortified ugali and plain *ugali*. (**B**) preference ranking of differently fortified *ugali*, different letters indicate significance between differently fortified *ugali*.

Caregiver evaluation of the five attributes of differently fortified *ugali* showed that all the sensory attributes, that may affect the acceptability of *ugali*, changed significantly with the addition of vegetables (Figure 6A). Using the plain *ugali* (blank) as a food vehicle, caregivers were able to detect differences between fortified *ugali* for color, odor, taste, granularity, and viscosity (*p* < 0.05). For all the attributes, ratings were undesirable for every fortified *ugali*, except Chinese anion stalk that is almost as identically desirable as the plain, unfortified *ugali*.

The preference ranking sequence of the seven flavors of *ugali* was: Chinese onion stalk-fortified, carrot fortified, cabbage fortified, blank (unfortified), broccoli fortified, bok choy fortified, and mushroom fortified (Figure 6A). However, except that the Chinese onion stalk-fortified *ugali* was significantly ranked higher than others (*p* < 0.05), ranking among other fortified *ugali* was not significant. Similar survey with a much larger population might resolve the differences between the preferences of different fortified *ugali* in the future.

## 4. Discussion

Five attributes of the plain and vegetable fortified *ugali*, color, odor, taste, granularity, and viscosity, were evaluated by assessors. Since people usually sensed color and odor before they further taste it, which includes taste, granularity and viscosity, we decided to discuss color and odor before taste, granularity and viscosity. Plain *ugali* exhibited a white, yet tint yellow color that easily distinguish it from any fortifications except Chinese onion stalk due to the light and similar color of the later. Bok choy, broccoli and cabbage all endowed *ugali* green color due to the chlorophyll in them with non-distinguishable extent to consumers. It seems that color alteration with tint colors does not distinguish themselves, as evidenced by the indistinguishable hedonic scale of *ugali* fortified with cabbage, carrot and Chinese onion stalk. Deep brown color, endowed by addition of bok choy, broccoli, and mushroom does not differentiate each other for consumers. Although the odor of carrot (endowed by the presence of linden ether) added *ugali* is the same to consumers as the plain *ugali*, the color between them tells the difference [33]. The odor of Chinese onion stalk (probably dipropyl disulfide and methyl propenyl disulfide) fortified *ugali* was statistically unidentifiable from that of plain *ugali* [34].

From the taste perspective, plain *ugali* and the Chinese onion stalk fortified one were unidentifiable from bok choy, cabbage, and carrot added ones. This is probably due to the rich flavor content in broccoli and mushroom that distinguished themselves [35,36]. Oral texture properties of *ugali* often limits the nutrition intake, especially in children, leading to protein-energy malnutrition [37]. The oral cavity sensation of granularity between the plain, carrot and Chinese onion stalk added *ugali* is the same to the potential consumers. This is probably due to the reason the highly rehydration process of freeze-dried carrot and onion stalk during *ugali* cooking that their texture differences between the plain *ugali* were eliminated [38]. The viscosity of plain *ugali* is indistinguishable from that of cabbage, carrot, and Chinese onion stalk fortified *ugali*. This is probably due to the absence of starch or second thickener in the fortified *ugali* system [39].

In summary, the Chinese onion stalk fortified *ugali* exhibited no significant sensory differences in color, odor, taste, granularity and viscosity compared with the plain, unfortified *ugali* for assessors. Both the caregiver evaluation and preferencing ranking indicates that assessors even preferred the Chinese onion stalk fortified *ugali* over the plain one. Another *Allium* vegetable, *Allium hookeri*, significantly increased its sourness, sweetness and umami taste after hot air-dry [40]. The overall preference of the freeze-dried Chinese onion stalk fortified *ugali* is thus highly likely due to the presence of sweetness and umami components in this vegetable, not serving to change the taste, but as a taste/flavor enhancer for the plain *ugali* [41,42,43].

This study is one of its kind in this area and will hopefully result in more studies with larger sample sizes investigating the possibility of improving the nutritional value of *ugali* through the use of vegetable pastes proposed in this study and more. However, although these vegetable pastes have been found to be a potential source of nutrition when added to *ugali*, one anticipated limitation to their use would be their availability in the different African countries where *ugali* is the staple meal. If already readily available in most if not all of these countries, then follow up in-country studies should be conducted to determine the acceptability of such mixtures by the actual local people. Although the side dishes commonly served with *ugali* in various countries might have some nutritional value, the results of this study show some promising results that in the areas where such high-nutritional value side dishes are not guaranteed, consumption of *ugali* fortified with the vegetable pastes reported in this paper could be an ideal option.

## 5. Conclusions

To increase the nutritional value of corn flour based *ugali*, specifically in vitamin A, carotenoids, and dietary fiber, freeze-dried powder of bok choy, broccoli, cabbage, carrot, Chinese onion stalk, and mushroom were added during *ugali* cooking. Fortification with these vegetables either changed the color, odor, or the taste, granularity, and viscosity that eventually lead to unfavorable assessor preferences. Except that the Chinese onion stalk fortified *ugali* cannot be distinguished from the plain, unfortified *ugali* while gained more favorable assessor preferences. This is probably due to the presence of umami components in the Chinese onion that serve as taste enhancer, thus renders *this* vegetable a promising candidate as vegetable supplement for the sub-Saharan Africa staple food. Meanwhile, on the basis of acceptance, bok choy, broccoli, cabbage and carrot variants had a comparable rating to that of plain *ugali*. Secondly, when the nutritional value was used as the basis, carrot, bok choy and onion stalk was found to considerably improve the micronutrient composition of *ugali*. Based on these findings, in addition to the Chinese onion stalk, bok choy and carrot options seem to be acceptable candidates for fortification which could be tested further in a follow up study involving a larger sample size.

## Figures and Tables

**Table 1:** Appellations of the same staple food in different African countries.

Appellation	Country
*Bugali*	Burundi, DR Congo, Rwanda
*Chima*	Mozambique
*Isitshwala*	Botswana
*Gari*	Gabon
*Kaunga*	Uganda
*Mealie pap*	Lesotho, South Africa
*Mutuku*	South Africa
*Ngima*	Kenya
*Nkima*	Kenya
*Nshima*	Malawi, Zambia
*Nsima*	Malawi, Zambia
*Oshifima*	Namibia
*Pap*	Namibia, South Africa
*Papa*	Lesotho, South Africa
*Shima*	Malawi
*Ubugali*	Rwanda
*Ugali*	Kenya, Malawi, Mozambique, Tanzania, Uganda
*Upswa*	Mozambique
*Vhuswa*	Venda
*Xima*	Mozambique

## Data Availability

Data are contained within the article.
